# Sex Differences in the Triad of Acquired Sensorineural Hearing Loss

**DOI:** 10.3390/ijms22158111

**Published:** 2021-07-28

**Authors:** Kuang-Hsu Lien, Chao-Hui Yang

**Affiliations:** 1 Department of Otolaryngology-Head & Neck Surgery, Chang Gung Memorial Hospital, Linkou Branch, College of Medicine, Chang Gung University, Taoyuan 33302, Taiwan; kathjj126@gmail.com; 2 Graduate Institute of Clinical Medical Sciences, College of Medicine, Chang Gung University, Taoyuan 33302, Taiwan; 3 Department of Otolaryngology-Head & Neck Surgery, Kaohsiung Chang Gung Memorial Hospital and Chang Gung University College of Medicine, Kaohsiung 83301, Taiwan

**Keywords:** acquired sensorineural hearing loss, noise trauma, ototoxicity, presbycusis, estrogen, cochlea, sexual dimorphism

## Abstract

The triad of noise-generated, drug-induced, and age-related hearing loss is the major cause of acquired sensorineural hearing loss (ASNHL) in modern society. Although these three forms of hearing loss display similar underlying mechanisms, detailed studies have revealed the presence of sex differences in the auditory system both in human and animal models of ASNHL. However, the sexual dimorphism of hearing varies among noise-induced hearing loss (NIHL), ototoxicity, and age-related hearing loss (ARHL). Importantly, estrogen may play an essential role in modulating the pathophysiological mechanisms in the cochlea and several reports have shown that the effects of hormone replacement therapy on hearing loss are complex. This review will summarize the clinical features of sex differences in ASNHL, compare the animal investigations of cochlear sexual dimorphism in response to the three insults, and address how estrogen affects the auditory organ at molecular levels.

## 1. Introduction

Hearing loss is the predominant health issue in recent decades because it places psychological and socioeconomic burdens on the world. According to World Health Organization (WHO), almost 6.1% of the world’s population has disabling hearing loss (about 432 million adults including 242 million males and 190 million females). Furthermore, it may rise to 630 million by 2030 and over 900 million by 2050 [1]. Acquired sensorineural hearing loss (ASNHL) is the most common type of hearing loss that includes noise-induced hearing loss (NIHL), ototoxicity, age-related hearing loss (ARHL), Meniere’s disease, and autoimmune-related hearing loss, as well as others. Among these, noise, ototoxic drugs, and aging account for the major contributing causes of ASNHL in modern society. The triad of ASHNL represents the damage of the auditory pathway in response to acute, subchronic, and chronic environmental insults [2].

While the clinical features of noise, drug, and age-related hearing loss had been well understood, recent studies have demonstrated the sex differences of hearing severity in the triad of clinical ASNHL patients and explored the mechanisms underlying the sexual dimorphism in the animal models. This review article will focus on sexual dimorphism in ASNHL from the clinical and basic perspectives, and will explore the available studies to elucidate the effect of sex hormones on the auditory organ.

## 2. Clinical Aspects of Sex Differences in Acquired Sensorineural Hearing Loss

### 2.1. NIHL

NIHL accounted for at least 16% of all disabling hearing loss and has a demanding societal cost [3]. The pattern of an audiogram in NIHL usually presents a notch at 4 kHz with a spread to the frequencies of 3 kHz and 6 kHz [4,5]. After prolonged exposure to noise, the lower frequencies at 0.5, 1, or 2 kHz may also be involved [6]. Several reports have noted significant sexual dimorphism in NIHL. In Norway, a series of studies showed that women exhibited better hearing preservation after adjusting noise exposure and occupational factors [7,8]. In the latest cross-sectional study including 1140 males and 1140 females in China, males had a higher risk of high-frequency hearing loss compared to females in equivalent noise exposure and age [9]. The largest meta-analysis of occupational NIHL also demonstrated that male workers had higher odds of experiencing high-frequency NIHL than female workers [10]. Another aspect of gender difference in NIHL was described in Chung’s study. They showed that males had a larger “ear effect” (right ear being more sensitive) in response to industrial noise exposure compared to females. In addition, females had better hearing than males after noise exposure in this study [11].

Apart from adults, NIHL in adolescents became a popular and crucial issue in recent years [12] but evidence of a sex difference in NIHL among adolescents is lacking. Several reports demonstrated the gender difference in their attitudes toward noise [13,14]. In 1997, Holmes et al. screened the hearing in 342 adolescents and 10.2% of males failed to pass at 6000 Hz in contrast to the 5% in females [15]. Males used firearms more frequently and a significant correlation was observed between failure at 6000 Hz and firearm use. Concerning the prevailing portable listening devices in recent decades, males had higher overall calculated exposure levels and chose higher levels of music in the quiet environment than females [14]. However, the hearing threshold at 4 kHz, which is most affected by noise, did not differ between males and females aged 12–19 in the South Korean population [16]. Additional work with longitudinal follow-up is necessary to explore whether recreational music has a differential impact on the hearing between male and female adolescents.

### 2.2. Ototoxicity

Drug ototoxicity is another main cause of ASNHL in modern society. Abundant evidence has shown that ototoxic agents were mainly transported from the strial vessels or diffused via the round window into the cochlea after intratympanic administration or systemic use [17,18,19]. Various targeted sites of the inner ear including hair cells, supporting cells, spiral ganglion cells, and the auditory nerve can be injured according to the properties of the drugs. Among these, hair cells are consistently the predominant vulnerable site [20]. The well-known ototoxic agents include aminoglycosides, loop diuretics, platinum-based chemotherapies, nonsteroidal anti-inflammatory drugs (NSAIDS), and so on. Although various ototoxic drugs were found, the gendered difference was only discovered in part due to the lack of research until recent decades. Franconi et al. summarized the gender difference in drug responses from the pharmacokinetics to pharmacodynamics aspects. They concluded that females were more likely to experience adverse drug reactions including ototoxic effects [21,22]. One cohort from Canada showed that the ototoxicity of an aminoglycoside antibiotic, amikacin, was associated with the female sex (females had a higher risk of ototoxicity than males) when treating patients with nontuberculous mycobacteria pulmonary disease [23]. In contrast, the ototoxicity risk of platinum-based chemotherapies such as cisplatin was higher in males [24,25,26,27]. The possible reason may attribute to the finding that some female cell lines are less sensitive to platinating agents than their male counterparts and may cause the phenotypic differences following cisplatin therapy [28]. However, some studies reported that platinum-based chemotherapies did not exhibit the gender difference in ototoxicity [29]. The sex difference of cisplatin ototoxicity still needs to be clarified due to the heterogeneous hearing results. 

### 2.3. ARHL

ARHL or presbycusis usually represents developing high-frequency hearing impairment and frequently occurs with poor speech discrimination [30]. According to the WHO estimation, approximately one-third of people have disabling hearing loss after 65 years old and half of those are individuals over 85 years old in the United States [31]. Hearing loss in the elderly is often associated with countless negative impacts on life including communication obstacles, isolation, late-life depression, cognitive decline, and so on [32,33,34,35]. For decades, substantial cross-sectional and longitudinal human studies in various regions described that ARHL had a higher prevalence in males than females [36,37,38,39,40,41,42]. Pearson et al. proposed that the hearing threshold declined twice as fast in men than in women at almost any frequencies and men had an earlier onset of hearing decline [36]. Although it was considered that males might experience more noise exposure than females, their data still showed similar hearing outcomes after adjusting the noise and occupational factors [8]. Meanwhile, similar results were noted in another study that found that the thresholds at 0.25 kHz and 8 kHz increased gradually every year, and men had significantly higher increasing rates than women [43]. Concerning the hearing thresholds at different frequencies, elderly males had higher hearing thresholds than females at higher frequencies during aging in longitudinal [36] and large cohort [44] studies. These studies demonstrated that hearing loss is more profound in elderly males than females.

In recent years, a growing literature has shown that hearing loss is a risk factor for dementia [45]. The less perception from the peripheral auditory system decreases the transduction of sound to the central cortical area and also reduces the neural activities and signals’ coding [32]. The treatment of hearing impairment could increase and maintain the cognitive reserve and prevent dementia as stated in the latest report of the Lancet Commission [35]. Although ARHL was identified as the most significant risk factor in dementia [46], the investigations of sex differences in the impact of ARHL on cognitive function were scarce. A study from Korea observed the association between hearing loss and cognitive impairment only in women aged 65 years and older [47], whereas recent research from a US national populational-based sample of adults aged 60 to 69 years old revealed that this association only appeared in males [48]. The discrepancy between the two studies may be due to the uncertain effect of gender differences in social networks [49,50,51]. While several factors could affect the sex differences in neurodegeneration [52], further studies to explore how ARHL affects cognition function in males and females would be helpful to determine whether hearing loss is a precocious sex-dependent indication of neurodegeneration.

### 2.4. Other Pathological Diseases Associated to ASNHL

Apart from NIHL, ototoxicity, and ARHL, Meniere’s disease and autoimmune inner ear disease are also associated with ASNHL. Meniere’s disease is characterized by fluctuating and progressive sensorineural hearing loss accompanied by episodic vertigo. Although the exact causes of Meniere’s disease are not clear, endolymphatic hydrops are likely causative of this disease [53]. Several reports had revealed a slight female preponderance in Meniere’s disease [54,55]. Those who had a lower estrogen level presented poor auditory function in postmenopausal patients with Meniere’s disease [56]. Autoimmune inner ear disease features fluctuating bilateral progressive sensorineural hearing loss within weeks or months, likely due to a consequence of antibodies from various conditions such as viral infection, trauma, and vascular injury that damaged the inner ear [57]. In addition, autoimmune inner ear disease commonly occurs in females [58], similar to the female predominance in systemic autoimmune diseases [59]. 

## 3. Animal Investigations of Sex Differences in Acquired Sensorineural Hearing Loss

The sex differences in ASNHL were also evident in subsequent animal studies [60,61,62]. Sexual dimorphism in the auditory system was observed in many species for decades. Nonmammalian species including frogs, praying mantises, birds, and so on were described in detail [63,64,65]. However, only mammals are summarized here given the anatomical and physiological similarities with humans.

A series of studies in sex differences regarding the mammalian auditory system including mice, rats, chinchillas, rhesus monkeys, spotted hyena, and sheep were conducted [60,66,67,68,69,70]. Of these, several strains of mice such as CBA/CaJ and C57BL/6J mice were considered as useful models and were extensively applied in most types of hearing loss studies because the auditory circumstance and potentially interacting factors can be carefully controlled [71]. Auditory brainstem response (ABR) and otoacoustic emission (OAE) were typically used in animal models as the objective auditory measurements.

There are sundry sexual facets that contribute to the roles of this dimorphism: genetic factors, anatomical differences, occupation type, employment status, and so on. Some studies reported that males possess a slightly longer cochlear length but this finding still lacked clinical data and pathophysiological evidence [72,73]. From the molecular aspects to the clinical presentations, the disparities of sex and sex-related hormones are of interest to scientists and clinicians. Therefore, studying the sexual dimorphism in animal models of the auditory system may help us develop treatments for hearing impairment based on different genders.

### 3.1. NIHL

Although several animal studies have reported the sex difference in NIHL, the results varied in different species or strains. Milon et al. demonstrated that after exposure to 2 h of octave-band noise, female B6CBAF1/J mice had a significantly lower compound threshold shift and reduced permanent threshold shift compared to control male mice. However, no significant difference in hair cell counts and inner hair cell synapse counts between the two groups was noted [60]. Another study found that after exposure to 100 dB SPL broadband noise, there was no difference in the ABR threshold but a significant effect on the frequency–sex interaction in CBA/CaJ mice was noted. In addition, females had more excitatory synapses of immunolabeling in the ventral cochlear nucleus at the lower frequency and less at the higher frequency [74]. This result was consistent with McFadden et al. who emphasized that female chinchillas had less low-frequency hearing loss than males but exhibited greater hearing loss at 16 kHz. Meanwhile, less hair cell loss in female chinchillas was noted [69,75]. However, in Willott’s study, female C57BL/6J mice lost more outer hair cells than ovariectomized female or male mice after exposure to nightly moderately intense augmented acoustic environments [76]. This opposite result may be attributed to the specific characteristic of C57BL/6J mice regarding elevated ABR thresholds of higher frequencies at 3 months of age and this trait may induce the interaction of ARHL and NIHL [62].

### 3.2. Ototoxicity

Sex differences in ototoxicity are also a widely discussed topic. Various animal models were examined for further investigation due to inconsistent human observational studies as mentioned above. One study provided direct evidence that the female cisplatin group had more deteriorated OAE values than the male cisplatin group among the Wistar albino rats. Although ABR values did not show a significant difference, the female cisplatin group had more apoptotic spiral ganglion neurons [77]. One recent study reported that inconsistent hearing thresholds after cisplatin injection were observed in different strains of mice [78]. For example, the CBA/CaJ mice revealed no significant sex difference; the female C57BL/6J mice had higher threshold shifts than the males at 4 kHz and 16 kHz. In contrast, in BALB/cJ mice, males had higher threshold shifts than the females at 4 k, 8 k, and 12 kHz. Interestingly, no significant difference in hair cell counts between male and female mice was observed in this study. There are two possible reasons for the heterogeneous results. First, there may be different susceptibilities to ototoxicity in these strains. Second, the different aging rates in these strains induced by ARHL may interfere with the degree of ototoxicities. Thus, the clear mechanism still needs to be investigated.

Regarding aminoglycosides, one animal study demonstrated that male Long–Evans rats had poor OAE values compared to female rats after treatment with kanamycin [79]. In the same manner, another study found that female guinea pigs that received gentamicin had better ABR performance than both the males with the same dosage treatment and the lower dosage male controls [80]. The diverse results between clinical and animal studies may be due to different animal species and drug pharmacodynamics. 

### 3.3. ARHL

To understand the mechanism of sexual dimorphism in presbycusis, various animal models were conducted. CBA/CaJ mice experience progressive high-frequency hearing loss first and then gradually experience low-frequency loss. In addition, CBA/CaJ mice do not develop premature hearing loss, thus they are a suitable animal model for evaluating aging hearing [81,82]. When mice are growing older, the trend of dropped sex hormone levels mimics the trends for humans. One study reported that middle-aged and elderly male CBA mice had decreased OAE levels which indicated the outer hair cell dysfunction, while female mice levels only declined after menopause [61]. Another study examined both CBA/J and CBA/CaJ mice for the onset of ARHL and found that male mice had significantly poorer high-frequency thresholds than the females but not in C57BL/6J mice [76,82]. Subsequently, the *Ahl* gene was proposed as the reason to explain the trait of C57BL/6J mice in having a different result compared to CBA mice [76,83]. Overall, the structural cochlear changes including in spiral ganglion cell counts or strial capillary density in these animal models provided evidence of sex differences in auditory organs during aging [76,84,85].

## 4. How Could Hormones Influence Hearing in Molecular Aspects?

As the above observational and animal studies have shown that females have better hearing than males in several ASNHL cases, the main sex difference in hearing may attribute to the distinct sex hormones in the respective genders. Among the sex hormones, estrogen is the most important and widely discussed hormone in this field. Androgen is less discussed in the auditory system and its effect on ASNHL is still under investigation.

### 4.1. Estrogen in Auditory Function

Estrogen plays the most crucial role in this issue. Some animal and human studies have demonstrated that women may be protected against hearing loss because of estrogen and its signaling pathways [86,87]. Several clinical reports have observed that the level of estrogen and its derivatives positively influence OAE amplitudes and ABR wave latencies [88,89,90,91,92]. Women had subtle fluctuating auditory thresholds during their menstrual cycle phases and their best hearing thresholds were observed at the highest peak of estrogen level [92]. Meanwhile, a more extensive scale study including 1830 postmenopausal women found the association between hearing loss and the serum estradiol level [93]. These observational studies imply the protective effect of estrogen on hearing. According to Guimaraes et al., pre-menopausal CBA female mice have healthier outer hair cells than middle-aged males [61]. These results agreed with McFadden’s study that interpreted the better response of the human female outer hair cell system with the presence of estrogen and stated that masculinization may decrease the auditory response [94].

### 4.2. Potential Molecular Effects of Estrogen and Receptors on the Auditory System

Estrogen was regarded as neuroprotective and neurotrophic for the brain and presumably had protective effects on the auditory system [95]. A series of research studies to investigate the mechanism of estrogen in molecular aspects were conducted. One is Turner’s syndrome (TS), characterized by females with a partly or completely missing X chromosome and leading to ovarian dysgenesis accompanied by little or none of the endogenous estrogen production [96]. TS provided scientists the perspective on how estrogen regulates hearing as progressive sensorineural hearing loss was commonly noted in women with TS and also in the TS animal model [96,97].

**Figure 1:**
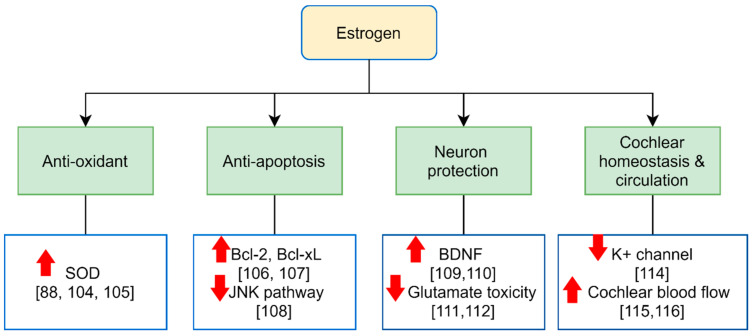
Overview of potential molecular effects of estrogen on the auditory system. Estrogen could enhance the expression of antioxidant SOD and reduce apoptosis by upregulating Bcl-2/Bcl-xL and inhibiting the JNK pathway. In addition, estrogen increases the BDNF in neurons and inhibits glutamate excitotoxicity. Estrogen could also help to modulate cochlear homeostasis and increase cochlear blood flow. Abbreviations: SOD = superoxide dismutase and BDNF = brain-derived neurotrophic factor.

Two main estrogen receptors (ERs) include estrogen-receptor-α (ERα) and estrogen-receptor-β (ERβ) [98]. Many transcriptional regulation mechanisms of estrogen receptors have been identified and mainly operate via direct DNA binding [99]. Both ERα and ERβ play crucial roles in the development and maintenance of normal sexual reproductive functions and modulate transcription by binding to estrogen response elements (ERE) [100]. In mice and rats, both ERα and ERβ can be detected in inner or outer hair cells, stria vascularis, spiral ganglion cells, vestibular cells, and in the central auditory system. However, one human study revealed that ERα was observed only in the spiral ganglion cells, whereas ERβ was observed in the stria vascularis of the human inner ear [101,102,103]. Therefore, how estrogen and receptors are involved in the functional well-being of the auditory system is still under investigation. The hypothetical molecular effects of estrogen on the inner ear is shown in Figure 1.

In hair cells, oxidative stress due to overproduction or insufficient detoxification of reactive oxygen species (ROS) during acoustic trauma, ototoxic drug exposure, and aging is the major contributor to cell death in ASNHL [2]. Estrogen could induce superoxide dismutase (SOD) expression in the blood and brain to increase plasma total antioxidant capacity and provide neuroprotection [104,105]. Therefore, it was speculated that estrogen would increase the antioxidant enzymes in hair cells to preserve hearing function [88]. In addition, estrogen can inhibit apoptosis via upregulating neuronal Bcl-2 and Bcl-xL [106,107]. In fact, a previous study has shown that estradiol protects the cochlea against gentamicin ototoxicity through the inhibition of the JNK (a pro-apoptotic) pathway [108]. Spiral ganglion neuron (SGN) loss is another cochlear pathology following hair cell death in ASNHL [2]. The SGN function is preserved by the brain-derived neurotrophic factor (BDNF) which could be enhanced by the ERβ agonist [109,110]. Furthermore, estrogen could protect the cortical neurons and auditory midbrain against glutamate excitotoxicity [111,112] which also plays an important role in the cochlear synaptopathy during ASNHL [113].

The cochlear lateral wall, which consists of a spiral ligament and stria vascularis, is also under estrogen modulation. Stria vascularis is important in the homeostasis of the water and blood circulation in thecochlea. There are several ion channels distributing stria vascularis and the K^+^ channel is influenced by estrogen levels [114]. Meanwhile, estrogen activates various downstream pathways to modulate the PI3K signaling and then enhance the nitric oxide production which can dilate vessels and increase the cochlear blood flow [115,116]. Therefore, estrogen may play an important role in maintaining hearing and balance. For example, estrogen levels correlated with auditory and vestibular function in postmenopausal patients with Meniere’s disease [56] because lower estrogen may be involved in the microcirculatory disturbance and endolymphatic hydrops of the inner ear [117].

Another type of orphan nuclear receptors that mimic the ERs is the estrogen-related receptors (ERRs). ERRs have sequence similarities with ERs and share transcription targets with ERs. The ERRs exhibit neuroprotection and modulate cell apoptosis in many target organs via regulating the ROS system or mitochondrial function and may also have roles in maintaining hearing function [118,119,120]. The mice study supported the hypothesis, demonstrating worse hearing performance in ERR gamma knockout mice [119]. 

### 4.3. The Role of Estrogen on Sex Differences in ASNHL

As estrogen seems to have protective effects on the auditory system in the molecular aspects, it is still unclear whether estrogen could account for the sex differences in the triad of ASNHL. For NIHL, Meltser et al. demonstrated that ERβ knockout mice had temporary hearing loss after acoustic trauma, whereas ERα knockout mice and wild-type mice did not [110]. In addition, the ERβ agonist treatment could reduce the temporary threshold shift. In female rats after ovariectomy, noise exposure can also cause more significant damage [121]. These results may partially explain the better hearing in females in response to noise observed in the clinic but the direct effect of estrogen on the auditory system in NIHL needs further elucidation.

In contrast to the possible protective effect of estrogen on the cochlea in NIHL, whether estrogen plays a role in ototoxicity is still under investigation. Low estrogen may be associated with the decreased distortion product OAE and increased ABR thresholds in ovariectomized rats after cisplatin treatment [122]. Conversely, estrogen could protect against gentamicin-induced outer hair cell death by inhibiting the JNK signal pathway in the organ of Corti explants [108]. Further exploration is needed to elucidate the effect of estrogen on the cochlea when exposed to ototoxic drugs. 

Regarding ARHL, the fluorescence intensities of ERα and ERβ were decreased in both sexes when aging and elderly female mice still had higher ERβ levels in spiral ganglion cells, vestibular hair cells, dark cells, and vestibular ganglion cells than males. In contrast, ERα level showed a gender difference only in spiral ganglion cells [102]. These findings strengthen the hypothesis that ERβ has a positive effect on hearing in ARHL. Interestingly, young female mice had a stronger fluorescence intensity of ERα than males did in the cerebral cortex. In addition, the higher ERα mRNA level was present in female mice but ERβ did not show a significant difference between young female and male mice [123]. This may account for the fact that young female mice had shorter ABR latencies and a larger amplitude than young male mice [124]. Further studies regarding hormone therapy for ARHL have demonstrated how estrogen affects ARHL which we will discuss in the “The Effect of Hormone Therapy on Hearing” section.

### 4.4. Androgen in Auditory Function

Androgen is another dominant sex hormone that regulates the development of male characteristics, strengthens muscle mass, and increases energy and libido. Androgen is mainly produced in the testicles in males but a part of androgen is synthesized in the ovaries and adrenal glands [125]. In contrast to the known effects of estrogen on hearing function, little is known about how androgen might influence hearing. In addition, whether androgen receptors (AR) are expressed in the inner ear of vertebrates is not clear. One of the well-established model systems for studying the neural and hormonal mechanisms among vertebrates is fish [126]. The distribution of AR mRNA expression in the inner ear of teleost fish supports the possible role of androgen as the modulator for the auditory system [127]. However, the specific role of AR in the inner ear still needs further investigation.

The previous animal study had shown that testosterone in serum increased neural thresholds in females in a frequency-specific way [128]. From a clinical perspective, the polycystic ovarian syndrome women with higher testosterone (one of the most potent androgen) presented significantly higher hearing thresholds at higher frequencies than controls. However, the above studies only included female participants and the luteinizing hormone, insulin, and other hormones could also influence their results [129,130,131]. In contrast, male patients with hypogonadism disorders such as Kallmann syndrome or Cogan syndrome were reported with sensorineural hearing loss but their hearing loss mostly has been attributed to genetic mutations rather than to testosterone deficiency only [132]. Although there is a lack of direct clinical evidence of testosterone and hearing loss, some studies considered that testosterone might have a negative impact on hearing because higher testosterone could lower the OAE amplitudes [66]. However, hyperandrogenism did not affect OAE or the medial olivocochlear reflex response in adult females [133]. As a result, whether androgen plays an essential role in ASNHL is still unclear. One recent report revealed that AR inhibition protected against cochlear injuries in kanamycin-induced hearing loss in rats [134]. Further investigations are needed to elucidate the role of androgen in the auditory system. 

## 5. The Effect of Hormone Therapy on Hearing

Even though the benefits of hearing protection from ERs were evident, the clinical usage of estrogen to protect or treat ASNHL remains difficult to validate. From a therapeutic perspective, estrogen regulates many physiological functions in the whole-body system such as cardiac, gastrointestinal, nervous, and respiratory systems rather than hearing alone. There is more evidence regarding the interactions between sex hormones and the function of the inner ear, especially in the mechanism of hearing impairment and balance disorders in elderly females and pregnant women [135]. Particularly, a lower level of serum possibly impedes hearing sensitivity in postmenopausal women [93]. Intrinsic estrogen or estrogen therapy might slow down the hearing loss in aging females [136,137]. These reports implied that estrogen is one of the key ways to preserve hearing in the aging human, especially for aging postmenopausal women. 

For decades, hormone-replacement-therapy (HRT) users are the most available group to evaluate how estrogen influences hearing in the clinical aspect. It is challenging to compare and interpret because HRT regimens vary in dosage, composition, duration, and initiation with regards to the onset of menopause [138]. Currently, the studies regarding HRT and hearing have mainly focused on postmenopausal female ARHL. One cross-sectional study found that postmenopausal women using HRT had higher serum estradiol levels and better pure tone thresholds than non-HRT treatments but no detailed regimens were disclosed [93]. Another prospective case-control study including a total of 109 women indicated that estrogen supplementation helped delay hearing loss in postmenopausal women. Those who took 17β-estradiol only had significantly better hearing performance than the non-HRT control. Interestingly, the estrogen group also showed significantly better hearing than the group using the combined regimen containing 17β-estradiol and norethisterone acetate (one of the progestin derivatives) [137]. These reports imply that estrogen alone shows benefits for hearing, while progestin has a negative impact on hearing. 

Another case-control study included 124 postmenopausal women and found that progestin (as a component of HRT) resulted in poorer hearing, whereas estrogen alone showed no significant hearing difference but still with a better trend than the non-HRT control [139]. Their group then conducted the parallel animal study in peri-menopausal mice via the usage of HRT and the result was consistent with their previous human study [140]. Similarly, a large observational human study from Curhanet et al. that included 80,972 women was also in agreement with Guimaraes’s finding [141]. Therefore, to clarify the specific effect from long-term HRT, a study used ovariectomized mice to examine hearing after six months using estradiol, progestin, and estradiol accompanied by progestin and a placebo [142]. According to their results, the estradiol-treated mice presented with lower thresholds and higher amplitude values of ABR compared to other hormone treatments. Meanwhile, progestin-treated mice had decreased ABR thresholds. These results were also strengthened by their in vitro and in vivo studies, showing that a high gene expression of IGF-1R, which can regulate anti-apoptotic responses in inner ear cells, is only present in the estradiol group rather than other groups [142]. These clinical and animal studies help us to determine how HRT and estrogen influence hearing in ARHL.

Taken together, estrogen has a positive effect on hearing and has been proved in vitro and in vivo. In clinical practice, estrogen and its derivatives are predominantly used in HRT regimens. However, most HRTs are comprised of progestin and its derivatives which showed negative effects on hearing. This is characterized as the main reason the previous human studies showed inconsistent outcomes. As we are aware that estrogen operates in the hypothalamic–pituitary–gonadal axis, which functions not only in hearing but throughout the whole body, the adequate dose necessary to reach the therapeutic effect and avoid side effects on other organs still needs to be investigated. Therefore, aside from using systemic estrogen as a therapy, localized use may be the better choice to apply estrogen and its derivatives to improve hearing impairment. Some strategies for cochlear drug delivery such as nanoparticles, hydrogels, or micropumps can be the focus of future investigations [143,144].

## 6. Conclusions and Future Perspectives

Sex differences are important in the studies of translational neuroscience [145]. Although the mechanisms underlying the triad of ASNHL may be similar, we need to consider sexual dimorphism during the interpretation of results in clinical and basic hearing research [146,147,148,149]. From a clinical perspective, females exhibited better hearing than males during noise exposure and aging, while animal investigations only demonstrated better hearing in females in ARHL (Table 1). Conversely, sex differences in drug-related hearing loss are still uncertain. Several articles have revealed the potential protective effects of estrogen at molecular levels but the exact mechanisms of hearing preservation by estrogen in the auditory system are not totally elucidated. Lastly, the evidence of estrogen to protect hearing mainly focuses on ARHL in postmenopausal woman and the effect of hormone therapy on the auditory organ is still unclear. In the current era of translation research and personalized medicine, future basic and clinical investigations to elucidate the sex differences in the cochlea are essential to help to develop personalized therapeutic strategies against ASNHL [148,150,151].

Looking ahead, the study of stem cells and gene therapy in recent years provides new directions for the development of ASNHL treatment in the future decades [152]. Although regeneration of hair cells seems promising in animal models [153], previous studies have revealed that men and women might respond differently to regenerative medicine therapies [154]. Therefore, sex differences must be considered before entering clinical trials. In addition, we also must consider gender-specific strategies in the era of gene editing (CRISPR-Cas9) [155]. The usage of gene therapy to upregulate the expression of ERs in the inner ear may be a potential therapeutic option to improve hearing for ASNHL in the future [156]. 

## Figures and Tables

**Table 1:** Summary of sex differences in the triad of ASNHL.

	Noise (NIHL)	Drugs (Ototoxicity)	Age (ARHL)
Clinical aspects	Females had better hearing [7,8,9,10,11]	**Aminoglycosides:** females had a higher risk [23] **Cisplatin:** males had a higher risk [24,25,26,27] or no gender difference [29]	Males had a higher prevalence [36,37,38,39,40,42,43]
Animal investigations	B6CBAF1/J mice: females had a reduced permanent threshold shift [60] CBA/CaJ mice: no sex difference [74] Chincillas: females had less hair cell loss [75] C57BL/6J mice: females had more hair cell loss [76]	**Cisplatin:** Wistar albino rats: females had more apoptotic spiral ganglion neurons [77] CBA/CaJ mice: no sex difference [78] C57BL/6J mice: females had higher threshold shifts [78] BALB/cJ mice: males had higher threshold shifts [78] **Aminoglycosides:** females had better OAE in the Long–Evans rats [79] and ABR in guinea pigs [80]	CBA mice: females had better hearing during aging [61] CBA/J and CBA/CaJ mice: males had higher high- frequency ABR thresholds in late-onset ARHL [82]
Estrogen effect	ERβ agonist reduced the temporary threshold shift after acoustic trauma in mice [110]	**Cisplatin:** low estrogen increased ABR thresholds [122] **Aminoglycosides:** estrogen protected against outer hair cell death [108]	Elderly female mice had higher ERβ levels than males and preserved better hearing function [102] Postmenopausal women who had a higher serum estradiol level had better pure tone thresholds [93] and estrogen supplementation helped delay hearing loss [137]
